# Kinetics and Energetics of Intramolecular Electron Transfer in Single-Point Labeled TUPS-Cytochrome *c* Derivatives

**DOI:** 10.3390/molecules26226976

**Published:** 2021-11-18

**Authors:** Petro Khoroshyy, Katalin Tenger, Rita V. Chertkova, Olga V. Bocharova, Mikhail P. Kirpichnikov, Natalia Borovok, Géza I. Groma, Dmitry A. Dolgikh, Alexander B. Kotlyar, László Zimányi

**Affiliations:** 1 Institute of Biophysics, Biological Research Centre, Temesvári Körút 62, H-6726 Szeged, Hungary; khoroshyy@gmail.com (P.K.); tenger.katalin@gmail.com (K.T.); groma.geza@brc.hu (G.I.G.); 2 Institute of Organic Chemistry and Biochemistry, Czech Academy of Science, Flemingovo Náměstí 542/2, 16000 Prague, Czech Republic; 3 Shemyakin-Ovchinnikov Institute of Bioorganic Chemistry, Russian Academy of Sciences, Miklukho-Maklaya 16/10, 117997 Moscow, Russia; cherita@inbox.ru (R.V.C.); o.bocharova@gmail.com (O.V.B.); kirpichnikov@inbox.ru (M.P.K.); dolgikh@nmr.ru (D.A.D.); 4 Biology Department, Lomonosov Moscow State University, Leninskie Gory 1/12, 119899 Moscow, Russia; 5 Department of Biochemistry and Molecular Biology, George S. Wise Faculty of Life Sciences, Tel Aviv University, Ramat Aviv, Tel Aviv 69978, Israel; natalbor@tauex.tau.ac.il (N.B.); s2shak@tauex.tau.ac.il (A.B.K.)

**Keywords:** cytochrome *c*, intramolecular electron transfer, TUPS, time-resolved spectroscopy, triplet excited state

## Abstract

Electron transfer within and between proteins is a fundamental biological phenomenon, in which efficiency depends on several physical parameters. We have engineered a number of horse heart cytochrome *c* single-point mutants with cysteine substitutions at various positions of the protein surface. To these cysteines, as well as to several native lysine side chains, the photoinduced redox label 8-thiouredopyrene-1,3,6-trisulfonate (TUPS) was covalently attached. The long-lived, low potential triplet excited state of TUPS, generated with high quantum efficiency, serves as an electron donor to the oxidized heme *c*. The rates of the forward (from the label to the heme) and the reverse (from the reduced heme back to the oxidized label) electron transfer reactions were obtained from multichannel and single wavelength flash photolysis absorption kinetic experiments. The electronic coupling term and the reorganization energy for electron transfer in this system were estimated from temperature-dependent experiments and compared with calculated parameters using the crystal and the solution NMR structure of the protein. These results together with the observation of multiexponential kinetics strongly support earlier conclusions that the flexible arm connecting TUPS to the protein allows several shortcut routes for the electron involving through space jumps between the label and the protein surface.

## 1. Introduction

Electron transfer involving various metabolites, external electron sources, and redox cofactors of proteins is a fundamental process in all domains of life on Earth. A major focus of research in biological electron transfer has been directed towards understanding how the distance that separates redox active centers in proteins and DNA and the molecular structure of the separation medium influence the electron transfer rates, which must be fast enough for physiologically relevant processes. Early studies of electron transfer in the photosynthetic reaction center [1] yielded simple exponential dependence of electron transfer rates on the separation distance. These data were interpreted with a model where the protein matrix was treated as a homogeneous barrier to tunneling. In contrast, other accumulated data [2,3] revealed that distant donor–acceptor electronic coupling in proteins depended on the secondary and tertiary structure as well as the side chain composition of the intervening polypeptide matrix. According to the latter viewpoint, pathways might exist through electron transfer proteins that would facilitate the flow of electrons between distant sites. The homogeneous barrier model was later refined to take into consideration the packing density distribution between the electron donor and acceptor regions [4]. Both models are properly parametrized to explain the observation that proteins conduct electrons better than, for example, water. In artificial chemical systems where the donor and acceptor groups are usually connected by a rigid molecular chain, packing density is less meaningful and pathways are better defined. In proteins, minor variations of the atomic structure due to thermal fluctuations at ambient temperatures will lead to continuous alterations in best pathways and the packing density.

Examination of the electron transfer mechanisms in proteins requires high time-resolution spectral measurements. Chemical modification with ruthenium complexes has allowed investigators to also examine, in non-photosynthetic proteins, the dependence of the electron transfer rate on the distance separating two, natively occurring and chemically introduced, redox centers [5,6]. However, the efficiency of this method is low, as only about 1% of the total protein population can be perturbed. An alternative method to study electron transfer reactions in proteins is based on the photochemistry of the photoactive label thiouredopyrene-trisulfonate (TUPS). Long lifetime, high yield of the excited triplet state, and appropriate redox properties make the dye useful for initiation and analysis of electron transfer reactions in chemical and biological systems [7,8]. The advantage of this system for electron transfer studies is its high efficiency; more than 20% of the protein molecules undergo intramolecular reduction in a single pulse. The high yield of photoreduction enables estimation of intramolecular electron transfer rates with a high level of reliability and accuracy. The redox properties and the bifunctional nature (oxidant and reductant) of TUPS have been discussed in detail in our earlier publication [9].

Mitochondrial cytochrome *c* is a relatively small, globular, heme containing redox protein. The distance from any point on the surface of the protein to the heme and the rather uniform protein packing are in the range where electron transfer can take place at an acceptable rate. Nevertheless, electron transfer between cytochrome *c* and its physiological partners, the cytochrome *bc_1_* complex, cytochrome *c* peroxidase, and cytochrome *c* oxidase, takes place after appropriate docking with the positively charged face of cytochrome *c* where the edge of the heme is exposed (albeit recessed) [10,11,12], assuring optimal electron transfer efficiency. Non-physiological electron transfer between the heme and other surface locations may still be relevant in biomimetic, bioelectronic, or biosensoric applications [13].

Cytochrome *c* was the first protein where the electron transfer between TUPS and the heme was demonstrated [7]. Since its introduction, TUPS has been used to initiate electron transfer in azurin [8], as well as between cytochrome *c* and cytochrome *c* oxidase [14,15]. Cytochrome *c* is particularly well suited for electron transfer studies. Its physico-chemical properties, including the reduction potential of the heme group, as well as its stability are well known. The protein has been used as a redox partner in electrode reactions and at biomimetic interfaces [16,17]. In our earlier work, we demonstrated that at least the reverse electron transfer between the reduced cytochrome *c* heme and the positive radical of TUPS may exhibit multiexponential kinetics. Molecular dynamics calculations provided a likely explanation for both the multiexponential behavior and for the distance dependence of the electron transfer rates. It appeared that the four-ring structure of the dye may occupy several positions close to the protein surface, stabilized by ionic interactions, and the electron may prefer a through space jump to or from the surface of the protein rather than the route following the covalent bonds connecting the dye to the labeled amino acid [18].

In the present work, we constructed a panel of horse heart cytochrome *c* mutants, each containing a cysteine residue on the surface of the protein. TUPS derivatives of these variants were obtained by labeling the corresponding cysteine residues. Several lysine residues on the surface of the protein were also labeled with an isothiocyanate derivative of the dye. We studied the kinetics of electron transfer between the heme and the TUPS- label, positioned at different sites on cytochrome *c* using the combined techniques of kinetic multichannel and single wavelength absorption spectroscopy. The temperature dependence of the electron transfer rate allowed the estimation of the electronic coupling term and the reorganization energy in several cytochrome-TUPS conjugates. Extrapolation to the maximal, barrierless rate yielded much higher values than those calculated from the atomic resolution structure of the protein, assuming electron transfer via the cysteine or lysine residue to which the dye is attached. These results further corroborate that rapid electron exchange between TUPS and the heme cofactor through the protein matrix can bypass the covalent link, making TUPS a useful tool to study intra- and interprotein electron transfer processes.

## 2. Results and Discussion

### 2.1. Preparation of Cytochrome c-TUPS Derivatives

**Figure 1:**
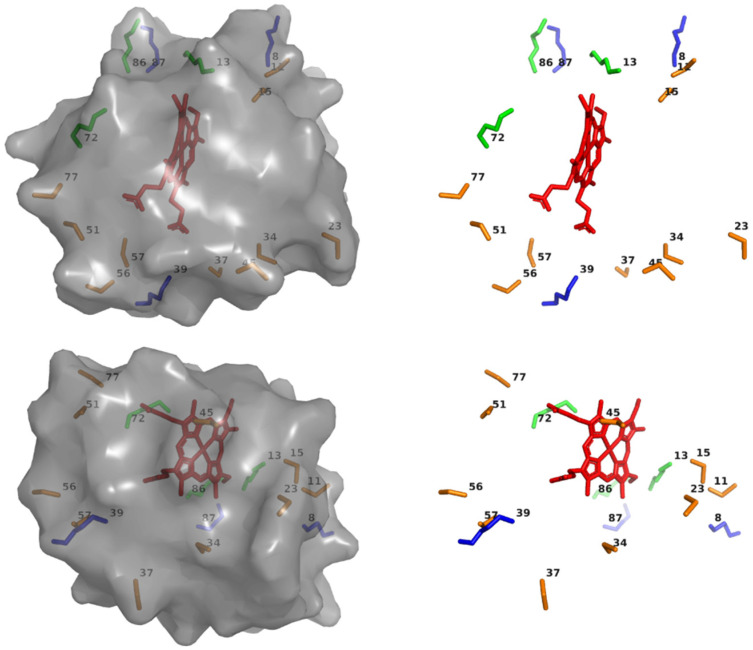
Structure of horse heart cytochrome *c*, with in silico mutated cysteines and native lysines that were labeled in this study by TUPS. Original structural file: 1HRC.pdb. Two different viewing angles (top and bottom row), with and without the protein surface (left and right) are shown. Color code: cysteines, gold; lysines that were also mutated to cysteines, blue; other lysines, green.

The selection of the location for the engineered cysteine residues was based on three criteria: the non-charged nature of the residues to be substituted, the accessibility of the residues from the water phase, and noninvolvement in the interaction with cytochrome *c* oxidase. In addition, three out of the six lysine residues labeled by TUPS were also mutated to cysteines and labeled to study the effect of the linker on the electron transfer dynamics. Figure 1 shows the crystal structure of horse heart cytochrome *c* (1HRC.pdb), where the mutated amino acids replaced in silico by cysteines and also the labeled native lysines are highlighted.

### 2.2. Quenching of TUPS Triplet Excited State Must Be Taken into Consideration in the Analysis of the Electron Transfer

**Figure 2:**
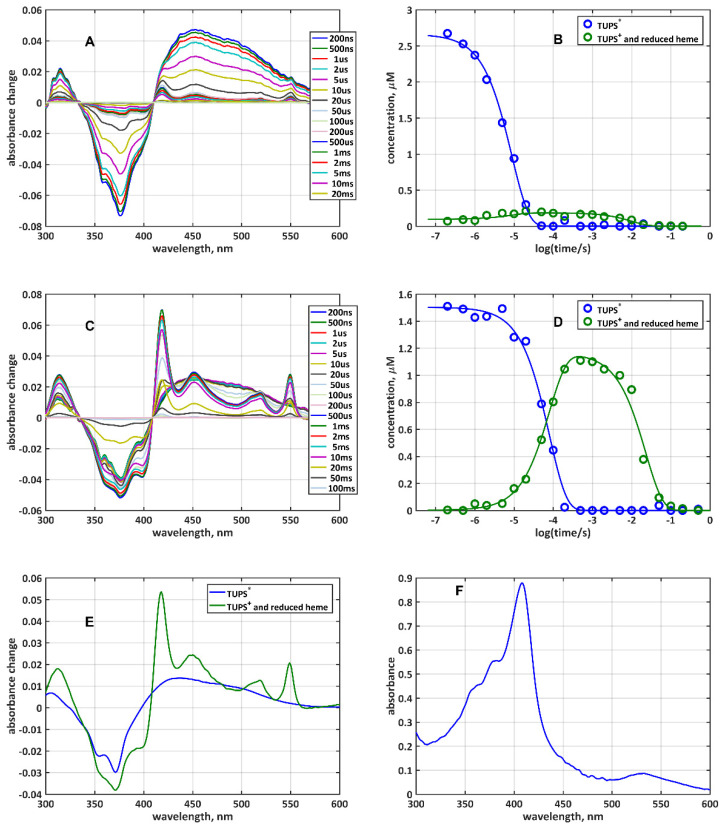
Kinetics of electron transfer between the dye and the heme in G23C-TUPS: Time-resolved difference spectra after laser flash excitation in the presence (**A**) and absence (**C**) of oxygen; (**B**,**D**) time-dependent concentrations of the {TUPS^T*^ + heme_ox_} and the {TUPS^+^ + heme_red_} species (symbols) and fit to Scheme 1 (lines). The rate coefficients obtained from the fit are: k_quench_ = 1.10 × 10^5^, k_forward_ = 3.84 × 10^3^, and k_reverse_ = 97.5 s^−1^ in the presence of O_2_, and k_quench_ = 2.84 × 10^3^, k_forward_ = 9.58 × 10^3^, and k_reverse_ = 43.7 s^−1^ in anaerobiosis; (**E**) base difference spectra used for the least-squares fit of the spectra in (**A**) and (**C**); (**F**) absorption spectrum of the G23C-TUPS sample before photoexcitation, with fully oxidized heme and characteristic TUPS bands in the 350–390 nm range.

**Scheme 1:**
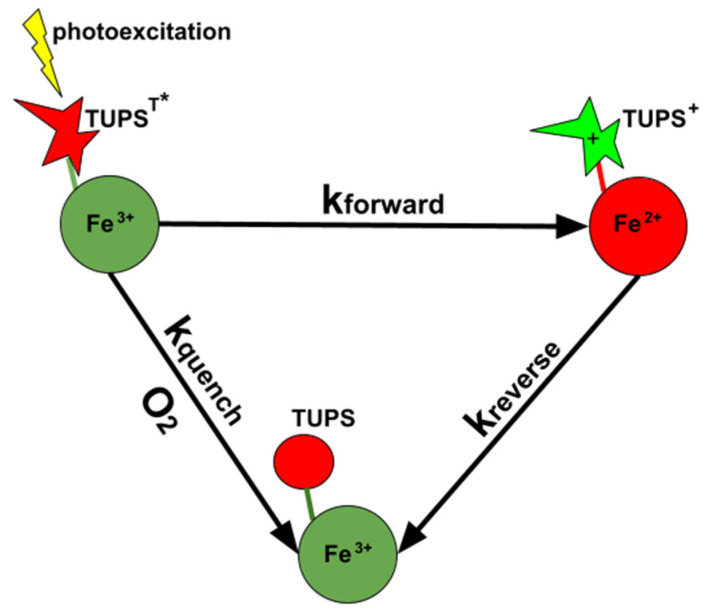
Kinetic model of the reactions following the photoexcitation in the TUPS-cytochrome *c* system.

Absorption kinetics experiments were carried out with TUPS-labeled G23C mutant without oxygen removal and in anaerobiosis. In the presence of oxygen, the initial triplet state decayed rapidly and only small amounts of TUPS^+^ and reduced heme were accumulated (Figure 2A). The amounts of the two forms, TUPS^T*^ and {TUPS^+^ plus heme_red_} (see Figure 2B), were calculated by least-squares fitting the difference spectra with the pure component difference spectra obtained earlier [9] (Figure 2E). The low yield of electron transfer is the result of TUPS^T*^ depletion by quenching, a process competing with the electron transfer from TUPS^T*^ to the oxidized heme. Under oxygen-free conditions, the lifetime of TUPS^T*^ is significantly longer and the dominant process is the electron transfer. We used the following model (depicted in Scheme 1) to analyze the kinetic data; k_forward_, k_reverse_, and k_quench_ respresent forward electron transfer, reverse electron transfer, and triplet quenching reactions, respectively.

**Figure 3:**
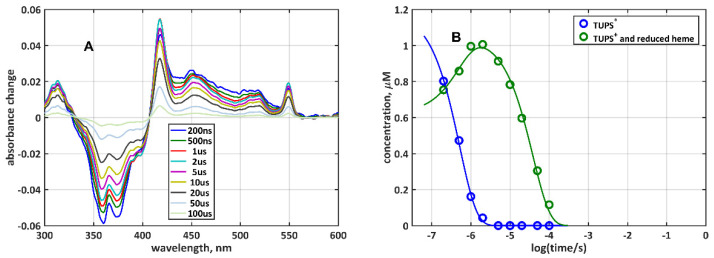
Kinetics of electron transfer between the dye and the heme in G77C-TUPS: (**A)** Time-resolved difference spectra; (**B)** time-dependent concentrations of the {TUPS^T*^ + heme_ox_} and the {TUPS^+^ + heme_red_} species (symbols), obtained by the least-squares fit of the spectra in (**A**) by the pure component spectra in Figure 2E, and fit to Scheme 1 (lines). The rate coefficients obtained from the fit are: k_quench_ = 1.24 × 10^6^, k_forward_ = 6.79 × 10^5^, and k_reverse_ = 2.59 × 10^4^ s^−1^.

The fit of this model to the kinetics of the product formation and dissipation (symbols in Figure 2B,D and Figure 3B) is shown as lines, and yielded the rate coefficients for the TUPS triplet quenching and the forward and reverse electron transfer.

In cases where oxygen removal was sufficiently complete, the calculated electron transfer rates were not significantly different from the observed rates that can be obtained by simple exponential fitting of the rising and falling phases of the component kinetics.

### 2.3. The Instantaneous Light-Induced Appearance of the {TUPS^+^ + heme_red_} Species: Role of Solvated Electrons

For several TUPS label positions, in the first difference spectrum, taken with 200 ns delay time after the actinic laser flash, a substantial amount of the {TUPS^+^ + heme_red_} species was detected (Figure 3). Since further electron transfer from TUPS ^T*^ to heme_ox_ was subsequently observed at a slower rate, the instantaneous production of the reduced heme could not be the result of the intraprotein electron transfer. The data in Figure 3 could be adequately fitted by Scheme 1, assuming that at time zero the initial concentration of {TUPS^+^ + heme_red_} was >0. One explanation could be the production of TUPS^+^ and solvated electrons [18,19,20,21,22] by the laser flash, followed by reduction of the heme by the solvated electrons. The instantaneous appearance of {TUPS^+^ + heme_red_} was typically observed in samples (V11C, A15C, A51C, and G77C) where the forward and reverse intraprotein electron transfers were fast, presumably due to the short distance between the solvent-exposed TUPS and the heme, so that solvated electrons could also be released near the heme cofactor.

### 2.4. Determination of the Coupling Terms and Reorganization Energies for Electron Transfer from Temperature Dependent Experiments

The rate coefficient of non-adiabatic electron transfer is described by Marcus theory [23,24] as:

(1) $$k=\sqrt{\frac{4\pi^{3}}{h^{2}\lambda k_{B}T}}H_{DA}^{2}\exp\left(-\frac{{\left(\Delta G+\lambda \right)}^{2}}{4\lambda k_{B}T}\right)$$

where *h* is Planck’s constant; *k*_B_ is Boltzmann constant; *T* is absolute temperature; Δ*G* is the midpoint reduction potential difference between the electron donor and acceptor pairs (TUPS^+^/TUPS^T*^, heme ox/red, and TUPS^+^/TUPS); λ is the reorganization energy; and *H*_DA_ is the donor–acceptor electronic coupling term. In a good approximation the pre-exponential term is an exponential function of the distance (geometric distance or connectivity) between the donor and acceptor, defining the dimensionless coupling term, *T*_DA_:

(2) $$\sqrt{\frac{4\pi^{3}}{h^{2}\lambda k_{B}T}}H_{DA}^{2}=10^{13}T_{DA}^{2}\;(1/\sec)$$

with

(3) $$T_{DA}=\exp\left(-½\beta \left(r-r_{0}\right)\right)$$

or

(4) $$T_{DA}=\prod_{i}\varepsilon_{i}$$

In the first, packing density model, *β* = 0.9ρ + 2.8(1 − ρ), with ρ being the packing density of the medium between the donor and acceptor and *r*_0_ is contact distance, usually taken as 3.6 Å. In the second, pathway model *ε*_i_ is the decay factor for the *i*th step along the best pathway connecting the donor and the acceptor, whose usual value is 0.6 for a covalent bond, 0.36 exp (−1.7(*r* − 2.8)) for a hydrogen bond, where r is the heteroatom distance in Å and 0.6 exp (−1.7(*r* − 1.4)) for a through space jump spreading a distance of *r* (in Å) [6,25].

Rearranging Equation (1) yields:

(5) logk+½logT=a(λ,HDA)+b(λ,ΔG)1T

with

(6) $$a\left(\lambda ,H_{DA}\right)=½\log(\frac{4\pi^{3}}{h^{2}k_{B}})-½\log(\lambda )+2\log H_{DA}$$

and

(7) $$b\left(\lambda ,\Delta G\right)=-0.434\frac{{\left(\Delta G+\lambda \right)}^{2}}{4\lambda k_{B}}$$

Measuring the electron transfer rate as a function of temperature allows the determination of two key parameters of Marcus theory: the reorganization energy and the electronic coupling term, provided that the driving force, the reduction potential difference of the electron donor and acceptor pairs is known. Using Equations (5)–(7) to calculate these parameters from the experimental data assumes that over a limited temperature range they can be considered constants or, to be more exact, negligibly temperature dependent as compared with the exponential 1/*T* dependence. Such limited and here neglected temperature dependence may arise from the thermal fluctuation of the protein structure and the surrounding medium. 

Hence, from the linear fit of the plot of log(*k*) + ½log(*T*) as a function of 1/*T* one can estimate the reorganization energy, λ, and the electronic coupling term, *H*_DA_, using the known values of the midpoint reduction potentials: −0.90 eV for TUPS^+^/TUPS^T*^, 0.22 eV for heme ox/red, 0.88 eV for TUPS^+^/TUPS [20,21]. Previously it was shown that the reduction potentials of cytochrome *c* with TUPS bound to different lysine side chains (including those reported in this study) agreed within experimental error (20 mV) with that of the unlabeled protein [7].

**Figure 4:**
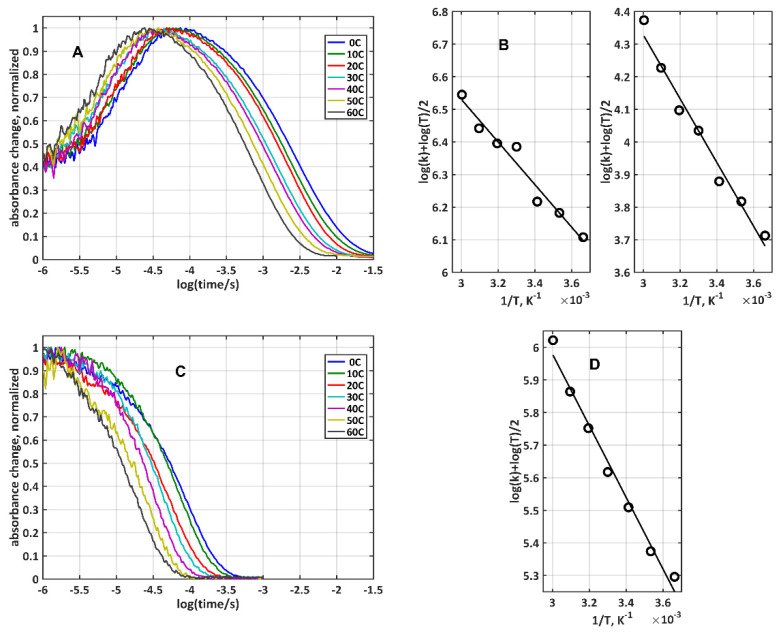
Kinetics of electron transfer measured at 7 different temperatures as ΔA_550_–ΔA_562_ with TUPS covalently bound to K8 (**A**) or K13 (**C**); only one of the electron transfer steps is resolved for the “fast” K13-TUPS sample. Single exponential fit to the rising and decaying phases (only one exponential for K13-TUPS) yielded apparent rate coefficients in (**B**,**D**) (symbols), with fit to Equation (5) as lines.

We have measured the electron transfer kinetics after photoexcitation of TUPS covalently bound to six different surface lysine residues: K8, K13, K39, K72, K86, and K87. Absorption change signals were recorded at 550 and 562 nm, the former corresponding to the maximum of the alpha band of the reduced heme *c* and the latter to the isosbestic point for the reduced minus oxidized difference spectrum of heme, thereby providing the appropriate signal to subtract the contribution of the TUPS^T*^ absorption. Data were recorded at 0, 10, 20, 30, 40, 50, and 60 °C. Characteristic ΔA_550_–ΔA_562_ traces for slow electron transfer with the K8-labeled and for fast electron transfer with the K13-labeled cytochrome *c* derivatives are shown in Figure 4A,C. Figure 4B,D show the plots corresponding to Equation (5). Rate coefficients shown in Figure 4B,D were obtained by fitting the kinetic traces by 2 exponentials (K8-TUPS, Figure 4A) or a single exponential (K13-TUPS, Figure 4C). A more refined analysis, with multiexponential fit of the kinetic traces is presented in the Supplementary Material.

From the quadratic equation for λ (Equation (7)) two values of reorganization energy and with Equation (6) the corresponding values of *H*_DA_ have been calculated (Table 1). A comparison of the reorganization energies with the free energy changes, Δ*G*, shows that the first set of solutions would mean electron transfer in the normal region (i.e., λ > |Δ*G*|) for the TUPS-cytochrome *c* system. The second set of solutions falls in the inverted region. The electronic coupling term is systematically smaller, reflecting less efficient coupling for the reverse electron transfer. The reorganization energy is also systematically smaller for the reverse electron transfer. These differences are likely to be the result of different electronic orbitals of TUPS participating in the forward and reverse electron transfer processes.

The reorganization energy for heterogeneous electron transfer can be estimated according to the Marcus cross relation as the mean of the self-exchange reorganization energies of the participating redox pairs:

(8) $$\lambda =½\left(\lambda_{1,1}+\lambda_{2,2}\right)$$

The self-exchange reorganization energy, *λ*_1,1_ for Fe(III/II) cytochrome *c*, was calculated as 1.04 eV [24], also consistent with the numerous values obtained for cytochrome *c* in electrode reactions [26]. On the one hand, although the self-exchange reorganization energy, *λ*_2,2_, for TUPS is not known, the second solution of the quadratic formula for *λ* (Table 1) yields values that would imply unrealistically low values for TUPS in solution (*λ*_2,2_). On the other hand, the first solution yields values indicating that electron transfer between the heme and TUPS takes place in the Marcus normal region.

### 2.5. Electron Transfer Routes between TUPS and the Heme: Maximal Rate from Experiment and Model Calculations

**Figure 5:**
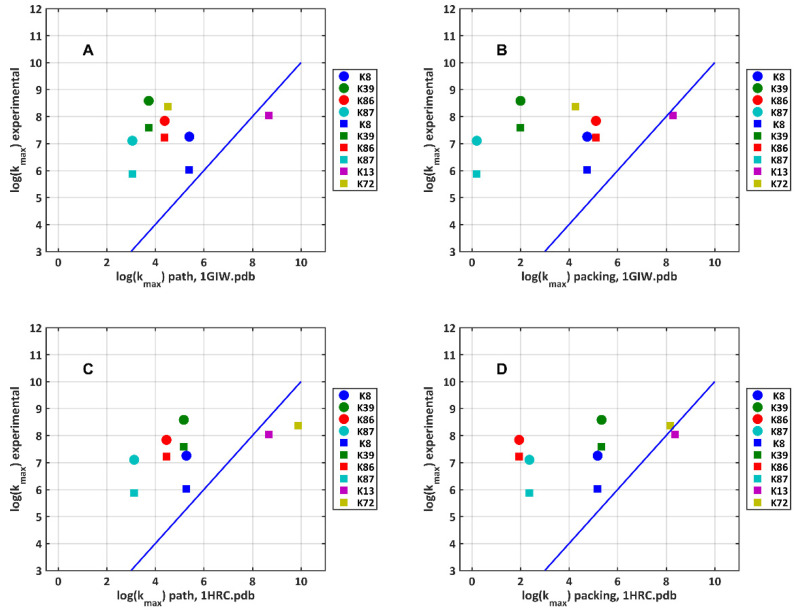
Comparison of the experimentally determined maximal electron transfer rates with the calculated maximal rates using two atomic resolution structural models of horse heart cytochrome *c*. Circles and squares represent forward and reverse electron transfer, respectively. Lines indicate equality between experimental and theoretical rates, to guide the eye: (**A**) Solution (NMR) structure used in the calculation with the pathway model; (**B**) solution structure, packing density model; (**C**) crystal (X-ray) structure used in the calculation, pathway model; (**D**) crystal structure, packing density model.

From Equation (1), one can obtain the maximal electron transfer rate (extrapolated to infinite temperature) between TUPS bound to different lysine side chains and the heme. The maximal rates extrapolated from the experiments for both the forward and the reverse electron transfer vary only within about two orders of magnitude despite the very different distances between the heme and the labeled lysine. For those four positions where the forward electron transfer could experimentally be resolved, the maximal rate for the forward electron transfer is about an order of magnitude higher than the reverse (Figure 5). We used the program HARLEM and either the crystal structure (1HRC.pdb) or the solution NMR structure (1GIW.pdb) of horse heart cytochrome *c* to calculate the optimal electron transfer pathways (pathway model, [27]) and the parameters of the packing density model [4] for the electron transfer between the epsilon N atom of the lysine residues and the edge of the π electron (ring) system of heme. The maximal rate provided by the model calculations is in most cases significantly smaller than the extrapolated maximal rate from the experiments (Figure 5). This is despite the fact that several covalent bonds connecting the lysine’s nitrogen with the ring structure of TUPS, which further extend the pathway and should decrease the calculated rate, were not taken into consideration. These results yield further support to our published model [18] that assumes that TUPS, connected with a flexible linker through the side chain of the labeled lysine or cysteine residues is capable of approaching the protein surface, assuring optimal access towards the heme via through space jump(s), leading to much faster electron transfer than when following the covalent link. This is corroborated by the fact that our recent bias-free, machine-learning-based multiexponential fit algorithm [28] yielded multiple, slightly different exponential components for the “slower” TUPS-lysine positions and also several cysteine-labeled positions (see Supplementary Material). Note that this advanced fit algorithm also confirmed that the data represent first-order kinetic steps rather than higher order or distributed kinetics processes. As before [18], we argue that TUPS can occupy several spatial positions relative to the protein surface and the heme which can support different electron transfer routes, short cutting the route following the covalent link.

Electron transfer parameters were calculated with the HARLEM program for two high resolution horse heart cytochrome *c* structures. Note that that the X-ray (1HRC.pdb) and the NMR (1GIW.pdb) structures are significantly different at certain surface positions due to the different side chain conformations. Since in our experiments surface-exposed side chains were labeled by TUPS, such differences in the corresponding side chain conformations yielded sometimes rather different calculated electron transfer parameters. Cases in point are the best pathway to the heme for lysine 72 or the packing densities to the heme for several surface lysine residues (Figure 5).

### 2.6. Electron Transfer Dynamics with Various TUPS-Labeled Cysteines

**Figure 6:**
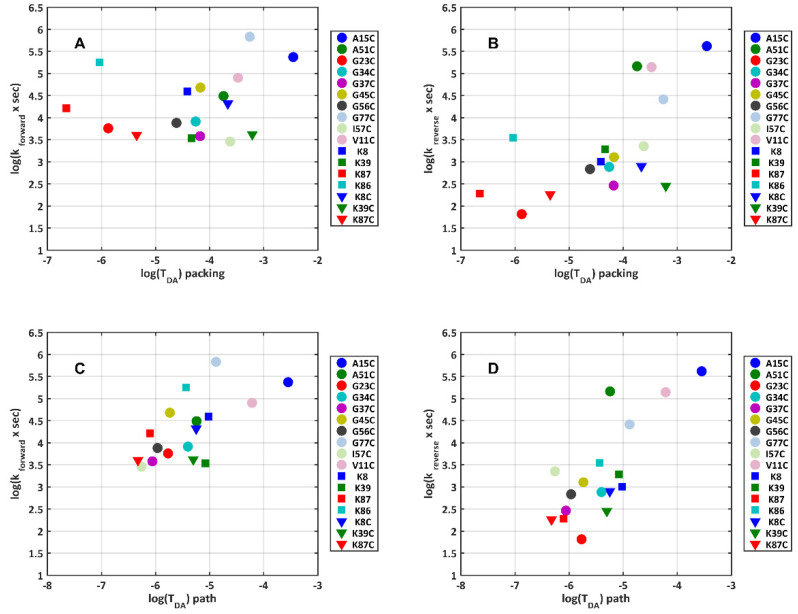
Forward and reverse electron transfer rates calculated using Scheme 1 from multichannel absorption kinetic measurements performed at room temperature. Rates are shown versus the dimensionless pathway or packing density coupling terms between the edge of the heme ring structure and the terminal atom of the labeled amino acid, calculated with HARLEM, using the structure 1HRC.pdb. (**A**,**B**) Forward and reverse rates, respectively, plotted versus the coupling term, *T*_DA_ calculated with the packing density model (Equation (3)); (**C**,**D**) forward and reverse rates, respectively, plotted versus *T*_DA_ calculated with the pathway model (Equation (4)).

In order to assure single label positions (i.e., homogeneous samples) and map the protein matrix in terms of electron transfer efficiency, we introduced site-specific cysteine residues, replacing either some of the lysine residues in the above experiments or other amino acids. Note that due to chemical differences in the label structures, cysteine labeling results in a link longer by one covalent bond, connecting the dye to the amino acid alpha carbon, than lysine labeling (Figure S2). Figure 6 shows the measured rate coefficients for the electron transfer between TUPS and the heme for 17 different label positions at room temperature, as a function of either the best pathway coupling term, or the packing density coupling term. These coupling terms are the dimensionless quantities, *T*_DA_, in Equations (3) and (4), calculated using HARLEM. The rate coefficients were obtained by fitting Scheme 1 to the multichannel spectroscopic data (as in Figure 2 and Figure 3). The path and packing coupling terms were calculated between the edge of the heme ring structure and the terminal atom of the labeled side chain (i.e., without the link from there to the TUPS ring structure). Assuming that the midpoint reduction potentials and the outer sphere reorganization energies for the TUPS forms do not significantly depend on the label position, the variations in the exponential term in Equation (1) can be neglected and the dependence of the rate coefficients on the label position should be controlled by the term *T*_DA_^2^. Although there is a tendency of faster experimental rates with higher coupling values, especially when calculated from the pathway model, the correlation is weak, and the rate data strongly scatter. This is again in agreement with our explanation that the flexible link connecting the dye to the protein allows the dye to approach the heme and exchange electrons with it faster than it would be possible from the point of the dye’s covalent attachment to the protein. Note that the electronic coupling term based on the packing density model is more sensitive to the conformation of the surface side chain than that based on the pathway model (Figure S3). This difference results in more horizontal scatter of the data points in Figure 6A,B than in Figure 6C,D.

## 3. Materials and Methods

### 3.1. Chemicals

Most chemicals were purchased from Sigma-Aldrich (Saint Louis, MO, USA) and AppliChem (Darmstadt, Germany); 1-isothiocyanatopyrene-3,6,8-trisulfonate (IPTS) was purchased from Lambda Fluorescence (Graz, Austria). Distilled water was additionally purified on a Milli-Q system (Millipore, Burlington, MA, USA).

### 3.2. Construction of Mutant Genes of Cytochrome c

Recombinant cytochrome *c* genes with single cysteine substitutions in 13 various positions: V11C, A15C, G23C, G34C, G37C, G45C, A51C, G56C, I57C, G77C, K8C, K39C, and K87C were obtained from the wild-type horse heart cytochrome *c* gene using site-directed mutagenesis with the Quick-Change Site-Direct Mutagenesis Kit (Stratagene, CA, USA), as described previously [29,30,31]. The nucleotide sequences of mutant genes in the plasmid DNA were determined on an ABI Prism 3100-Avant Genetic Analyzer (Applied Biosystems, Beverly, MA, USA).

### 3.3. Expression, Isolation, and Purification of Cytochrome c Mutants

Expression of the mutant genes of cytochrome *c* was performed in the JM-109 strain of *E. coli*, as described previously [31,32]. After the growth, cells were homogenized using a French press (Spectronic Instruments, Inc., Rochester, NY, USA) at high pressure with subsequent centrifugation at 95,000× *g*. Purification of the target proteins were performed on a BioLogic HR liquid chromatographic system (Bio-Rad, Hercules, CA, USA), according to the previously elaborated scheme [33]. The degree of protein purity was determined by absorption spectroscopy and SDS-PAGE electrophoresis. The fractions with A_409_/A_280_ ratio of 4.5:5.0 (corresponding to a purity of ≥95% for the substance commercially prepared by Sigma-Aldrich, Saint Louis, MO, USA) were dialyzed three times against 10 mM ammonium carbonate buffer (pH 7.9), and lyophilized. All stages of isolation and purification of proteins were controlled by electrophoresis in 12% Tris-tricine PAGE under denaturing conditions [34]. Concentrations of mutant proteins were determined by absorption spectroscopy at 409 nm (ε = 1.06 × 10^5^ M^−1^ cm^−1^) [35].

### 3.4. Preparation of TUPS-Modified Cytochrome c Derivatives

Surface-exposed lysine and cysteine side chains of cytochrome *c* were labeled with TUPS, according to published procedures [7,18]. Briefly, lysines were labeled by incubating chromatographically purified cytochrome *c* with IPTS at 38 °C for 48 h in 0.5 M KCl at pH 7.5 and the labeled proteins were separated from the excess dye by size-exclusion chromatography. The lysine-labeled TUPS-cytochrome derivatives were separated by ion-exchange HPLC [7]. The thiol-specific TUPS derivatives were prepared by incubating IPTS with cystamine at pH 9.0 for 6 h at room temperature. Cytochrome *c* with an engineered single surface cysteine was reduced with 5 mM dithiotreitol (DTT) for one hour to break possible interprotein disulfide bonds. The protein was separated from DTT by size-exclusion chromatography and incubated with an 8-fold excess of TUPS-cystamine, as described in [18]. The unbound dye was separated from the labeled protein by size-exclusion chromatography.

### 3.5. Kinetic Spectroscopy

A combination of a multichannel and a single wavelength detector in the same light path [18,36,37] was used to obtain time-resolved difference spectra (with high-spectral resolution and moderate temporal sampling intervals) and absorption kinetic traces at selected wavelengths with fine temporal sampling. TUPS-labeled cytochrome *c* was excited by the third harmonic of a Nd-YAG laser (Continuum Surelite-II). The energy density of the 355 nm, 5 ns laser pulse at the sample was 20 mJ/cm^2^. A continuous white measuring light from a 35 W Hamamatsu high pressure Xe lamp, passing the sample perpendicular to the exciting laser light, was dispersed by a Jobin-Yvon spectrograph (HR320). Two different detectors could be selected by a switching mirror. Multichannel spectroscopy was performed on a gated optical multichannel analyzer (Princeton Instruments IRY512) or an Andor iStar gated ICCD detector (Andor Technology, Belfast, UK) with 100 nanosecond time resolution. Difference spectra were collected at several delays per decade, by averaging 10–20 scans. The spectrum taken at 1 sec delay served as the reference. Single wavelength absorption kinetic traces were measured with a 20 MHz sampling rate at 550 nm, and reference traces at 562 nm were subtracted, to obtain the kinetics of heme reduction and reoxidation. Noise suppression of these traces was achieved by averaging over windows of logarithmically increasing width. All measurements were done at 20 °C, in 10 mM HEPES buffer at pH 7.5. Single wavelength kinetic measurements on lysine-labeled samples were performed in the 0–60 °C temperature range. The TUPS-cytochrome *c* concentration was between 10 and 20 μM, the protein was oxidized by substoichiometric amounts of cytochrome *c* oxidase, and oxygen removal was achieved by adding 20 mM glucose, 100 μg/mL glucose oxidase, and 10 μg/mL catalase. 

### 3.6. Data Analysis and Modeling

Data analysis, including the fit of the data matrices by the base spectra of the pure forms, the multiexponential fit of the kinetic traces, as well as fitting the kinetics by the reaction scheme (Scheme 1) were performed by programs written in «Matlab» (The Math Works, Natick, MA, USA). Electron transfer parameters were calculated from the atomic resolution structures of cytochrome *c* using the program HARLEM [https://crete.chem.cmu.edu/index.php/software/2-uncategorised/18-harlem, accessed on 15 November 2021].

## 4. Conclusions

The advantages of using TUPS to study electron transfer lie in the relatively easy chemistry to achieve singly labeled pure protein samples, the high quantum efficiency of the excited triplet state (TUPS^T*^) production, and the low reduction potential of the triplet. On the one hand, the flexibility of the linker connecting TUPS to amino acids (lysine or cysteine) allows the mobility of the dye to the extent that it is not possible to assign the entry point of the electron to an exact location on the protein surface. On the other hand, our experiments and calculations show that electron injection by TUPS into the heme can be much faster than expected from the distance or connectivity based on the position of the amino acid to which the dye is attached. This is a clear advantage when TUPS is used to initiate intraprotein or interprotein electron transfer in more complex systems than cytochrome *c* alone. Indeed, electron transfer was successfully characterized between internal cofactors by TUPS-labeled cytochrome *c* in complex with cytochrome *c* oxidase, and the flavin cofactors in the more complex TUPS-labeled enzymes, cytochrome P450 reductase (CPR) and the reductase domain of neuronal nitric oxide synthase (nNOS) [14,15,38].

## Figures and Tables

**Table 1:** Experimentally determined reorganization energies and electronic coupling terms for the electron transfer between TUPS and the heme in cytochrome *c*-TUPS conjugates.

		1st Solution		2nd Solution		
		λ, eV	H_DA_, eV	λ, eV	H_DA_, eV	ΔG, eV
K8	forward	2.18	3.96 × 10^−5^	0.57	2.84 × 10^−5^	−1.12
	reverse	1.86	9.19 × 10^−6^	0.23	5.48 × 10^−6^	−0.66
K39	forward	2.54	1.90 × 10^−4^	0.49	1.26 × 10^−4^	−1.12
	reverse	2.17	5.78 × 10^−5^	0.20	3.19 × 10^−5^	−0.66
K86	forward	2.27	7.85 × 10^−5^	0.55	5.52 × 10^−5^	−1.12
	reverse	1.96	3.72 × 10^−5^	0.22	2.16 × 10^−5^	−0.66
K87	forward	2.20	3.34 × 10^−5^	0.57	2.38 × 10^−5^	−1.12
	reverse	1.87	7.76 × 10^−6^	0.23	4.61 × 10^−6^	−0.66
K13	reverse	1.97	9.54 × 10^−5^	0.22	5.52 × 10^−5^	−0.66
K72	reverse	1.86	1.36 × 10^−4^	0.23	8.10 × 10^−5^	−0.66

## Data Availability

The data presented in this study are available on request from the corresponding author.

## Supplementary Materials

The following are available online, Figure S1: Temperature dependence of the amplitudes and rate coefficients obtained from the multiexponential fit of the kinetic traces measured on the K8-TUPS sample, Figure S2: Structure of TUPS as linked to lysine or cysteine side chain, Figure S3: Comparison of the dimensionless coupling terms calculated with HARLEM using the pathway and the packing density models for various native and engineered side chains of horse heart cytochrome *c*, Table S1: Experimentally determined reorganization energies and electronic coupling terms for the electron transfer between the heme and TUPS bound to lysine 8, in various presumed conformations.

## Sample Availability

Samples of the compounds are not available from the authors.
