# Supplementary material for: Kinetics and Energetics of Intramolecular Electron Transfer in Single-Point Labeled TUPS-Cytochrome c Derivatives
Source: Molecules. 2021 Nov 18;26(22):6976. doi: 10.3390/molecules26226976 (PMC8621336; doi:10.3390/molecules26226976)
Supplement: Supplementary file 1 [file molecules-26-06976-s001.zip › molecules-1468374-supplementary.pdf]

Supplementary material

Single wavelength absorption kinetic traces measured at various temperatures on different lysine- labeled TUPS-cytochrome *c* derivatives were analyzed by our recent machine-learning algorithm-based fitting program [28], https://github.com/groma-geza/FOkin accessed on 17 November 2021. The program determined that the kinetics reflect first order reactions (i.e. discrete exponential processes and no distributed kinetics) and suggested the number of exponential terms individually for the different samples and temperatures. Based on this output a fine tuning of the multiexponential fit was performed where the temperature variation of the exponential amplitudes was limited for a given sample to reflect separate conformational sub-populations. The temperature dependence of the obtained rate coefficients was used to calculate the reorganization energies and electronic coupling terms for the sub-populations. These values agreed reasonably well with the overall values as shown in Table I. For illustration, results for K8-TUPS are shown in Figure S1, and Table S1.

The rate coefficients measured at 20°C for the 3 reverse electron transfer components differ only by about an order of magnitude. Considering that the electron transfer rate depends very strongly on the distance and the packing density between the redox partners, small differences in the conformation of the TUPS-cytochrome system can result in the observed variations of the rate coefficient for the same physical process.

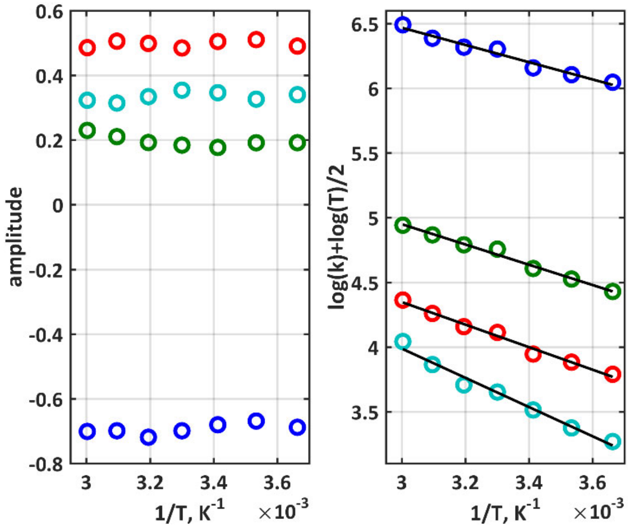

Figure S1. Temperature dependence of the amplitudes and rate coefficients obtained from the multiexponential fit of the kinetic traces measured on the K8-TUPS sample. Blue: forward electron transfer; green, red and cyan: reverse electron transfer.

Table S1. Experimentally determined reorganization energies and electronic coupling terms for the electron transfer between the heme and TUPS bound to lysine 8, in various presumed conformations. As an example the rate coefficients measured at 20°C are also listed.

		1st solution		2nd solution			
		λ, eV	H_{DA}, eV	λ, eV	H_{DA}, eV	relative amplitude	rate coefficient at 20 ^{o}C, s^{-1}
K8	forward	2.20	3.87×10^{-5}	0.57	2.76×10^{-5}		8.40×10^{4}
	reverse 1	1.68	9.42×10^{-6}	0.26	5.90×10^{-6}	19 %	2.36×10^{3}
	reverse 2	1.76	6.46×10^{-6}	0.25	3.95×10^{-6}	48 %	5.15×10^{2}
	reverse 3	2.00	1.07×10^{-5}	0.22	6.18×10^{-6}	33 %	1.90×10^{2}

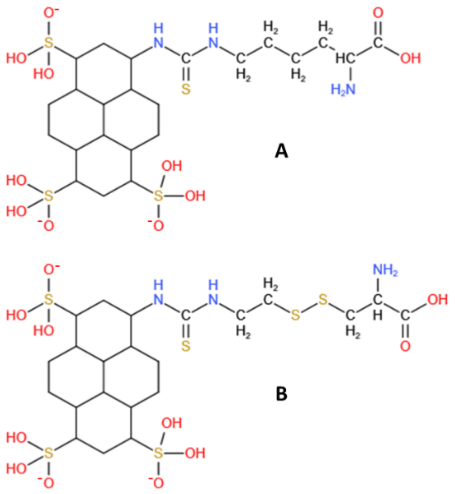

Figure S2. Structure of TUPS as linked to lysine (**A**) or cysteine (**B**) side chain.

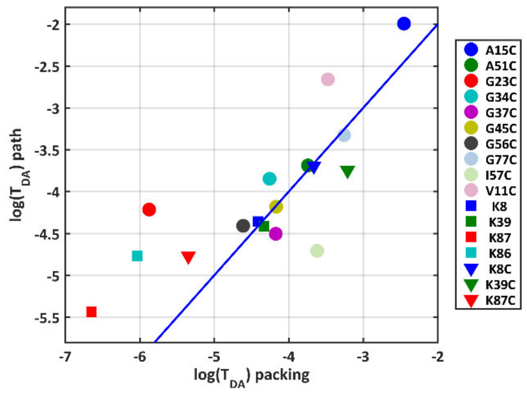

Figure S3. Comparison of the dimensionless coupling terms calculated with HARLEM using the pathway and the packing density models for various native and engineered side chains of horse heart cytochrome *c*. Blue line indicates equality between the two models, to guide the eye. Structure used: 1HRC.pdb.
